# Supplementary material for: Immunostaining protocol for infiltrating brain cancer spheroids for light-sheet imaging
Source: PLoS One. 2023 Feb 9;18(2):e0281161. doi: 10.1371/journal.pone.0281161 (PMC9910650; doi:10.1371/journal.pone.0281161)
Supplement: S1 File — (PDF) [file pone.0281161.s001.pdf]

Immunostaining infiltrating spheroids as preparation for quantitative light-sheet imaging V.2

Benedicte Bjørknes[1], Oliver Emil Neye[1], Petra Hamerlik[2,3], Liselotte Jauffred[1]

[1]The Niels Bohr Institute, University of Copenhagen, Blegdamsvej 17, DK-2100 Copenhagen O, Denmark;
[2]Danish Cancer Society, Strandboulevarden 49, 2100 Copenhagen Denmark;
[3]Division of Cancer Sciences, University of Manchester, M13 9NT Manchester, United Kingdom

Liselotte Jauffred

VERSION 2

JAN 05, 2023

OPEN ACCESS

DOI: dx.doi.org/10.17504/protocols.io.eq2ly77krlx9/v2

Protocol Citation: Benedicte Bjørknes, Oliver Emil Neye, Petra Hamerlik, Liselotte Jauffred 2023. Immunostaining infiltrating spheroids as preparation for quantitative light-sheet imaging. **protocols.io** https://dx.doi.org/10.17504/protocols.io.eq2ly77krlx9/v2Version created by Liselotte Jauffred

MANUSCRIPT CITATION: PLOS2022 this protocol is associated with a PLOS ONE Lab Protocol Submission with the title: "Immunostaining protocol for infiltrating brain cancer spheroids for light-sheet imaging" with manuscript number of our PLOS ONE submission: PONE-D-22-31029

License: This is an open access protocol distributed under the terms of the Creative Commons Attribution License, which permits unrestricted use, distribution, and reproduction in any medium, provided the original author and source are credited

ABSTRACT

Although various in vivo and in vitro models for studying glioblastoma cell invasion has progressed the field, there is still a need for optimized procedures. In particular to reveal key features of glioblastoma biology and infiltrating growth. In this protocol, we present an approach using indirect immunofluorescence in a 3D human xenograft glioblastoma spheroid model embedded in a naturally derived extracellular matrix

ATTACHMENTS

protocol.pdf

Protocol status: Working
We use this protocol and it's working

Created: Jan 05, 2023

Last Modified: Jan 05, 2023

PROTOCOL integer ID: 74805

Keywords: glioblastoma, invasion assay, immunostaining, hydrogels, spheroids, tumorospheres, light-sheet microscopy

Immunostaining infiltrating spheroids as preparation for quantitative light-sheet imaging

Benedicte Bjørknes[1], Oliver Emil Neye[1], Petra Hamerlik[2,3], and Liselotte Jauffred[1,*]

[1]The Niels Bohr Institute, University of Copenhagen, Blegdamsvej 17, DK-2100 Copenhagen O, Denmark
[2]Danish Cancer Society, Strandboulevarden 49, 2100 Copenhagen Denmark
[3]Present: Division of Cancer Sciences, University of Manchester, M13 9NT Manchester, United Kingdom
[*]jauffred@nbi.dk

ABSTRACT

Although various in vivo and in vitro models for studying glioblastoma cell invasion has progressed the field, there is still a need for optimized procedures. In particular to reveal key features of glioblastoma biology and infiltrating growth. In this protocol, we present an approach using indirect immunofluorescence in a 3D human xenograft glioblastoma spheroid model embedded in a naturally derived extracellular matrix.

Introduction

With this protocol we hope for future studies into the gene expression profiles during different stages of cancer invasion. For this reason, parameters were chosen to maintain the structural integrity of the spheroid and extra-cellular matrix as well as to increase immunofluorescence signal intensity, depth, specificity, and homogeneity to allow for quantitative microscopy.

The protocol is optimized for a human xenograft glioblastoma model. However, the methodological approach is adaptable to other spheroid systems, as long as the experimenter consider the following tests prior to experimentation. First of all, to follow the procedure with/without both primary and secondary antibody as well as with/without the permabilization step. Secondly, to investigate the possibility of co-staining of several antibodies by doing the full immunostaining procedure mixing primary and secondary antibodies from different systems. In this way, cross-reactions or leakage between the antibodies is revealed.

Reagents and equipment

Cell culture of human xenograft glioblastoma

Glioblastoma tissue samples (T115) were obtained from patients undergoing resection for newly diagnosed or recurrent tumors in accordance with a protocol approved by the Central Scientific Ethics Committee of the Copenhagen University Hospital.

For cell culture of this human xenograft glioblastoma cell lines we used the following media containing the supplemental nutrients – as well as the basic nutrients – to support fastidious or mutant growth requirements.

Null medium 500 ml Neurobasal®-A Medium (Minus Phenol Red) (Invitrogen 12349-015)

Complete medium 500 ml null medium with the following supplements
10 ml 50× B27 supplement minus vitamin A (50X) (Invitrogen 12587010)
5 ml 100× Glutamax (Invitrogen 35050-038)
20 ng/ml EGF recombinant, 1 mg (R&D systems 236-EG-01M)
20 ng/ml FGF recombinant, 1 mg (R&D systems 4114-TC-01M)
5 mL Penicillin-Streptomycin (100 units/mL penicillin and 100 μg/mL streptomycin)

Cell culture of primary specimen

In parallel we used the primary glioblastoma cell line (U-87).

Null medium 500 ml Phosphate Buffered Saline (PBS)

Complete medium 500 ml Dulbecco's Modified Eagle's Medium (DMEM)
50 mL Fetal Bovine Serum (FBS)
5 mL Penicillin-Streptomycin (100 units/mL penicillin and 100 μg/mL streptomycin)

Surface-attachment reagents and equipment

35 mm culture dishes, high precision 1.5 coverslip, 14 mm glass (Mattek, P35G-0.170-14-C)
2-well culture insert (Ibidi) with a sticky underside (Ibidi 81176)
Countess® II FL Automated Cell Counter (Thermo Fisher)

Spheroid formation reagents and equipment

Corning® Matrigel® GFR Membrane Matrix (Merck 356231)
Corning® Costar® Ultra-Low Attachment Multiple Well Plate (Merck CLS7007-24EA)
Countess® II FL Automated Cell Counter (Thermo Fisher)
$-20°$cold sterile P100 filter tips
Fluorinated Ethylene Propylene (FEB) tube with an inner/outer diameter of 0.8/1.6 mm

Immunostaining

For the immunostaining procedure we used the following reagents.

Washing buffer Gibco® Phosphate Buffered Saline (PBS)

Fixation reagent 4% Para-formaldehyde in PBS

Blocking reagent Freshly mixed 5% Gibco® Fetal Bovine Serum (FBS) in PBS. Secondary Antibody host serum can be preferable to avoid unspecific interactions between secondary antibody and serum. We tried both and did not see any difference, so we decided to keep with FBS for simplicity

Permabilization reagent 0.5% Triton X-100 (TX-100) in PBS

Vimentin primary antibody solution Vimentin Antibody (280618) [Unconjugated] (Novus Biologicals MAB2105) diluted 1:50 in blocking reagent (5% FBS in PBS)

Vimentin secondary antibody solution NorthernLights 557-conjugated Goat Anti-Rat IgG Seconday Antibody (NL013) diluted 1:100 in blocking reagent (5% FBS in PBS)

Nuclear-staining reagent SYTO™13 Green Fluorescent Nucleic Acid Stain (5 mM Solution in DMSO) (Thermo Fisher Scientific S7575)

Procedure

To prepare human xenograft glioblastoma spheroids infiltrating their local environment for imaging, we first need to culture cells, form spheroids, and enable invasive growth before immunostaining.

Cell culture

1. Thaw 1 ml stock cells in heat bath at 37°

2. Add cells to 5 mL medium and spin for 4 min at 300 g

3. Remove supernatant

4. Resuspend the pellet in 5 mL medium (37 °C) in a 60 mm culture plate to ensure that the cells have time to overcome the stress before being diluted further (∼50% confluency)

5. Incubate for 24 hours (37°C, 5% CO_2, and 100% humidity)

6. Transfer cells to a small T-flask (T25) and add 3 mL extra medium

7. Incubate for 2-3 days (37°C, 5% CO_2, and 100% humidity) before experimentation

Gravitation-assisted spheroid formation

To promote spheroid formation the cells are seeded in ultra-low attachment plastic U-bottomed 96-well plates.

1. Move cells from T-flask to 15 mL conical tube
2. Wash the T-flask with null medium and add this to the tube; as to collect all cells
3. Spin for 4 min at 300 g
4. Aspirate supernatant and re-suspend the pellet in 1 mL medium
5. Split cells by gently pipetting up and down through a 200 μl pipette tip
6. Use the cell counter to prepare a solution with density of 2,500 cells/ml
7. In a 96-well plate, add the cells solution to a final volume of 200 μL pr. well; corresponding to 500 cells/well (or more dependent on cell line)
8. Incubate for 3 days (37°C, 5% CO_2, and 100% humidity)

These spheroids can be immunofluorescence stained in two ways: In liquid medium (following the procedure in section *Immunostaining surface-attached cells*) or after embedment in hydrogel as described in the following.

Invasion assay

This protocol is based on a protocol by Maria Vinci and co-workers[1]. We used a concentration of 50% Matrigel (v/v) in medium but this protocol can easily be changed for a higher or lower percentage of hydrogels. To examine the migration of spheroid cells through a hydrogel matrix, spheroids are embedded and incubated for an appropriate amount of time (7 hours - 24 hours).

1. Thaw Matrigel overnight by placing it in an ice bucket in the fridge (4°C)
2. Prior to use, remove the ice but keep the Matrigel in the fridge for another 30 min
3. Place the 96-well plate on ice
4. Gently remove 150 μL of medium from each well. NB: Minimize disturbance of the spheroid
5. Add 50 μL Matrigel to each well using ice-cold pipette tips. NB: Upright pipetting on top of the spheroids, while not touching the well and barely the medium
6. Take care not to create any bubbles. If bubbles appear, remove bubbles with sterile needles (or by inflation)
7. Repeat for all wells
8. Incubate the the 96-well plate for 1 hour (37°C, 5% CO_2, and 100% humidity) for solidification
9. Add another 100 μL medium on top of the gel
10. Repeat for all wells
11. Incubate up to 72 hours (37°C, 5% CO_2, and 100% humidity) for the spheroids to invade

Immunostaining spheroids through hydrogel

The cells are stained with sets of primary (attaching to the protein in question) and a secondary antibody (carrying the dye). This protocol is based on a protocol provided by the antibody manufacturer, Novus Biologicals, where all incubation times has been extended to allow diffusion through the hydrogel. The permabilization step (2) should only be included for antibodies known to tag intra-cellular proteins as, e.g., the intermediate filament vimentin.

1. Fixation
   (a) Aspirate excess media and wash 3$\times$ with 200 μL ice-cold PBS. Allow the PBS to diffuse through the hydrogel for 5 minutes between each wash. Remove all excess buffer after the final wash
   (b) Add 100 μL (1$\times$ volume-equivalent) fixation reagent and incubate (room temperature) for 20 min

(c) Aspirate fixation reagent. NB: As para-formaldehyde liquifies Matrigel, aspiration is difficult to do in one go without harming the sample. Instead add 125 μL washing buffer and aspirate 175 μL (containing diluted paraformaldehyde). Repeat this once again to remove the 100 μL fixation reagent

(d) Wash 2× with 200 μL washing buffer. NB: Plate can henceforth be stored (leaving 100 μL PBS on gel) at 4°C for up to 4 days

(e) Aspirate washing buffer

2. Permeabilization

   (a) Add 100 μL of permabilization reagent and incubate at room temperature for 10 min

   (b) Aspirate permabilization reagent and wash 3× with 200 μL (2× volume equivalents) washing buffer (2× 10 min followed by 1× 30 min). Remove excess washing buffer before continuing

3. Blocking

   (a) Add 200 μL blocking solution and incubate for 1.5 hours (room temperature) cover 96-well with Parafilm® to avoid excessive evaporation

   (b) Aspirate blocking solution

4. Primary antibody incubation

   (a) Add 200 μL of the primary antibody solution and incubate for 2 hours (room temperature) or overnight at 4°C

   (b) Aspirate primary antibody solution

   (c) Wash 3× with 200 μL (2× volume equivalents) of washing buffer (3× 20 min)

5. Secondary antibody incubation

   (a) Add 100 μL of the secondary antibody solution and incubate for 1.5 hours (room temperature). NB: To avoid photobleaching cover with tinfoil

   (b) Wash 3× with washing buffer (1× 20 min followed by 2× 10 min)

Nuclear staining through hydrogel

After immunostaining we complemented with nuclear stains. As the vimentin-associated secondary antibody had maximum (absorption/emission) of (557/574) nm wavelengths we chose a green SYTO™ stain, which stains DNA and also some RNA with (absorption/emission) peaks of (488/509) nm and (491/514) nm, respectively. Hence, primarily the nucleus will light up, but also the cytosol will be stained. To minimize bleaching, all incubation should be under tinfoil.

1. Thaw dye from freezer and mix 2:10,000 in PBS

2. Add 100 μL and incubate for 3 hours (room temperature)

3. Aspirate and wash 1× with 200 μL washing buffer

In our hands this dye (SYTO™13) stained specifically and with appropriate yield. However, we had less success with similar nuclear stains when we sampled the SYTO™ kit (Thermo Fisher S11340), as we often found too much unspecific labelling for our requirements. Therefore, some work must be done to find the optimal set antibodies and nuclear stains for the proteins in question.

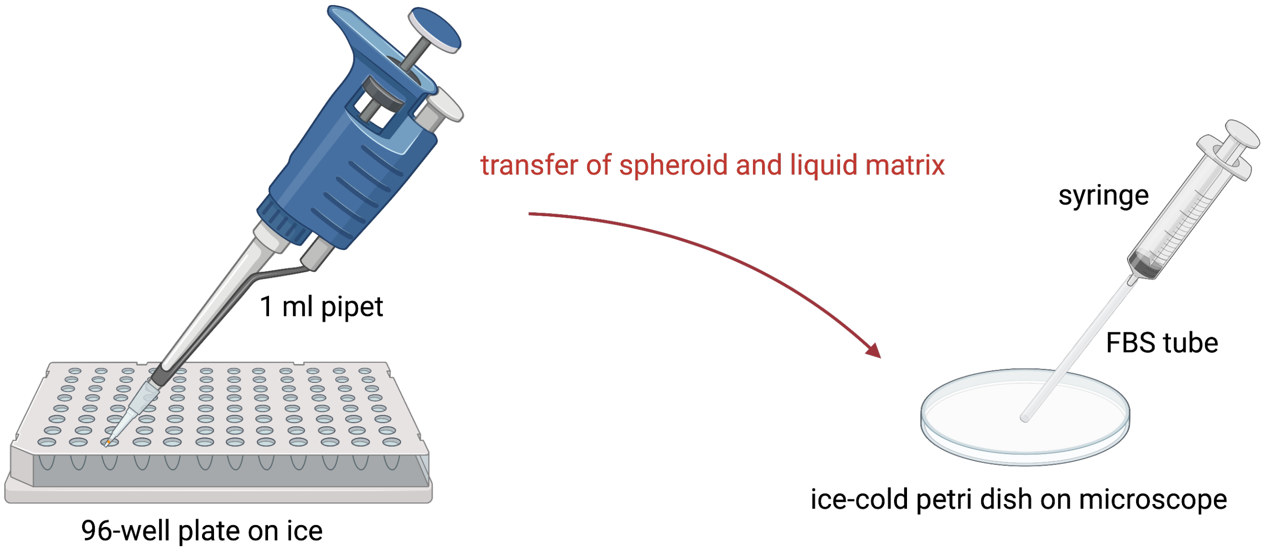

Figure 1. Two-step transfer of spheroids into the FEB tube prior to imaging. This sketch is created with BioRender.com.

Imaging spheroids in hydrogel

Spheroids in Matrigel that has invaded the local environment, can be moved after fixation and staining. However, this requires that the Matrigel is re-liquefied to allow the displacement of the spheroid to the appropriate imaging sample. For light-sheet imaging we followed these steps:

1. Place the the 96-well on ice to liquefy the hydrogel matrix
2. Gently aspirate the spheroid and hydrogel into the FEB tube by attaching the tube to a syringe. This part is tricky, so alternatively spheroids and matrix can be moved from the well to an ice-cold culture dish with a 1 ml pipette (to prevent damaging flow rates), before being aspirated into the tube under a microscope, as sketched in Figure 1. This alternative procedure allows for better localization of the spheroid (or spheroids) in the tube
3. Then the spheroid in the tube is ready for, e.g., light-sheet microscopy as described earlier[2]

We used an Aurora Airy Beam Light Sheet Imaging System[3–6], which is a selective/single plane illumination microscope equipped with two lasers for excitation of green (488 nm) and orange/red (561 nm) fluorescent molecule. The two objectives are placed very close together to match focus of detection (UMPlanFL N 20x/0.50 W) and illumination (UMPlanFL N 10x/0.30 W) objectives. For full imaging of spheroids (200 μm), stacks of 500 images with step size of 0.4 μm was collected with exposure times $<$150 ms and laser intensities $<$1.5% to avoid over-saturation.

Surface growth experiment

To test the antibody-staining and perform various checks, we used this more straight-forward procedure immunostaining for combination with wide-field fluorescence imaging.

1. Move cells from T-flask to 15 mL conical tube
2. Wash the T-flask with null medium and add this to the tube; as to collect all cells.
3. Spin for 4 min at 300 g
4. Aspirate supernatant and re-suspend the pellet in 1 mL medium
5. Split cells by gently pipetting up and down through a 200 μl pipette tip
6. Use the cell counter to prepare a solution with density of $5-7\times10^5$ cells/mL

7. In a 35 mm culture dish, add the cells solution in a final volume of 2 mL
8. Incubate for 24 hours (37°C, 5% CO_2, and 100% humidity)

Wound healing experiment

Alternatively, we performed a classical *wound* healing (or scratch) assay, followed by immunostaining and combined with wide-field fluorescence imaging.

1. Wash the T-flask with null medium and add this to the tube; as to collect all cells
2. Spin for 4 min at 300 g
3. Aspirate supernatant and re-suspend the pellet in 1 mL medium
4. Split cells by gently pipetting up and down through a 200 μl pipette tip
5. Use the cell counter to prepare a solution with density of $5 - 7 \times 10^5$ cells/mL
6. In a 35 mm culture dish, place the 2-well culture insert
7. Add cell solution to a final volume of 70 μL in each well
8. Incubate for 24 hours (37°C, 5% CO_2, and 100% humidity) to allow cells to settle and create a confluent layer
9. Remove culture insert to create a gap (i.e. *wound*) of 500 μm
10. Wash briefly with PBS or null medium
11. Add 2 mL fresh medium
12. Incubate for another 24 hours (37°C, 5% CO_2, and 100% humidity)

Immunostaining surface-attached cells

The cells are stained with sets of primary (attaching to the protein in question) and a secondary antibody (carrying the dye). This protocol is based on a protocols provided by the antibody manufacturer, Novus Biologicals. The permabilization step (2) should only be included for antibodies known to tag intra-cellular proteins as, e.g., the intermediate filament vimentin. This procedure can also be used for immunofluorescence-staining of spheroids in liquid culture.

1. Fixation
   (a) Aspirate excess media and wash with 200 μL ice-cold PBS
   (b) Add 100 μL (1$\times$ volume-equivalent) 4% Paraformaldehyde-PBS + 0.25% glyteraldehyde and incubate (room temperature) for 10 min
   (c) Wash with washing buffer. NB: Plate can henceforth be stored (leaving 100 μL PBS on gel) at 4°C for up to 4 days
   (d) Aspirate washing buffer
2. Permeabilization
   (a) Add 100 μL of permabilization reagent and incubate at room temperature for 5 min
   (b) Aspirate permabilization reagent and wash with 200 μL (2$\times$ volume equivalents) washing buffer. Leave for 5 min. Remove excess washing buffer before continuing
3. Blocking
   (a) Add 200 μL blocking solution and incubate for 1 hour (room temperature) cover 96-well with Parafilm® to avoid excessive evaporation
   (b) Aspirate blocking solution

4. Primary antibody incubation
   (a) Add 200 μL primary antibody solution and incubate for 1 hour (room temperature) or overnight at 4°C
   (b) Aspirate primary antibody solution
   (c) Wash 3× with 200 μL (2× volume equivalents) of washing buffer (3× 5 min)
5. Secondary antibody incubation
   (a) Add 100 μL of the secondary antibody solution and incubate for 1 hour (room temperature). NB: To avoid photobleaching cover with tinfoil
   (b) Wash 3× with washing buffer (3× 5 min)

This was combined with the procedure of nuclear-staining exactly as described in the section *Nuclear staining through hydrogel*.

Nuclear staining of surface-attached cells

After immunostaining we complemented with nuclear stains. As the vimentin-associated secondary antibody had maximum (absorption/emission) of (557/574) nm wavelengths we chose a green SYTO™ stain, which stains DNA and also some RNA with (absorption/emission) peaks of (488/509) nm and (491/514) nm, respectively. Hence, primarily the nucleus will light up, but also the cytosol will be stained. To minimize bleaching, all incubation should be under tinfoil.

1. Thaw dye from freezer and mix 2:10,000 in PBS
2. Add 100 μL and incubate for 2 hours (room temperature)
3. Aspirate and wash 1× with washing buffer

Imaging of surface-attached cells

We used a Nikon Eclipse Ti-E microscope system provided with a Lumencor SOLA Light Engine, to control the fluorescent intensity (20%) and a 40x oil objective. For vim (575/641 nm) detection we used a Texas red filter and for SYTO™13 (474/525 nm) detection a FITC filter. The exposure was 500 ms and the build-in image stitching algorithms of the Nikon software was applied. To minimize gradients over stitched images, the region of interest was restricted to 800 × 800 pixels.

Troubleshooting

Matrigel handling It is important that the Matrigel do not exceed 10 °C, since this is when it starts to solidify. As stated above, we used 4% para-formaldehyde in PBS for fixation. However, it is recognised that Matrigel can depolymerise after fixation with para-formaldehyde. It has therefore been suggested to add 0.25% glutaraldehyde alongside the 4% para-formaldehyde, as glutaraldehyde prevents depolymerisation and thus maintains the integrity of the 3D structure[7]. Unfortunately, glutaraldehyde introduces significant background fluorescence. So, to to minimise glutaraldehyde-induced autofluorescence, 0.1M glycine in PBS can be used (to react with unreacted aldehydes)[8] together with the reducing agent, sodium borohydride (NaBH4), to reduce double-bonds within aldehydes and ketones (unspecific redox reaction). It is worth mentioning that adding reducing agents could perturb antibody-antigen recognition and potentially cause other artifacts.

Optimizing specificity A protocol with a combined fixation (4% Para-formaldehyde) and permeabilization (1% Triton-X100) step has been suggested[9] to maximize antibody penetration depth in spheroids, when targeting surface proteins.

Another parameter to tune in case of low specificity, is the concentration of formaldehyde (fixative). There is a balance between using sufficient amounts of fixative to preserve cellular morphology without reducing the antigenicity[10].

Also, as antibodies targeting intracellular proteins require permeabilization of the cells' plasma membranes, we used Triton X-100 in a concentration of 0.2% diluted in PBS. However, insufficient permeabilization can hinder antibody penetration, while too high Triton X-100 concentration can lead to the loss- or displacement of the target protein[11,12]. We therefore suggest varying Triton X-100 concentrations between 0.1-1% to optimize for your protein of interest.

Co-staining with multiple antibodies For co-staining the antibodies should be immunostained sub-sequentially with a blocking step in between. Please note that after the first staining, the following procedures should be performed in the dark (i.e. under tinfoil). For the chosen pair of antibodies, potential cross-reactivity should be tested. For instance by doing the full immunostaining procedure and mixing primary and secondary antibodies from the two different systems.

Low levels of fluorescence For diagnostics, immunostain the spheroids while in liquid media following the protocol of section *Immunostaining surface-attached cells*. The result can be helpful when trying to tune the protocol of section *Immunostaining spheroids through hydrogel* for proper staining of your tissue sample.

Background fluorescence It is important to ensure adequate washing after antibody incubation to ensure specific binding of the antibodies to the target proteins. Adding more or longer washing steps could be beneficial in counteracting unspecific antibody binding.

Time taken

Immunostaining spheroids through hydrogel is estimated to take 8 hours and 40 minutes. Incubation overnight is recommended during the primary antibody staining thus splitting the procedure into a 5 hours and 30 minutes segment for day 1 and a 3 hours and 10 minutes segment for day 2. Fixation and permeabilization are both around 1 hour each. Blocking is 90 minutes. Primary antibody staining is either 2 hours or overnight followed by 1 hour of washing and secondary antibody staining is 2 hours and 10 minutes.

Anticipated results

3D fluorescent images of spheroids with finger-like protrusion for precise mapping of gene-expression differences between multiple cell types manipulated by different mechanisms. These mechanisms could be the effects of anticancer drugs, knockouts/knockdowns of specific proteins and so forth. Thus, the implications of adaptable immunofluorescence invasion protocol can enable the investigation of the proteins introduced here as well as other proteins and signalling pathways.

References

1. Vinci, M., Box, C. & Eccles, S. A. Three-dimensional (3D) tumor spheroid invasion assay. *J. visualized experiments : JoVE* e52686, DOI: 10.3791/52686 (2015).
2. Niora, M. *et al.* Head-to-Head Comparison of the Penetration Efficiency of Lipid-Based Nanoparticles into Tumor Spheroids. *ACS Omega* **5**, 21162–21171, DOI: 10.1021/acsomega.0c02879 (2020).
3. Voie, A. H., Burns, D. H. & Spelman, F. A. Orthogonal-plane fluorescence optical sectioning: Three-dimensional imaging of macroscopic biological specimens. *J. Microsc.* **170**, 229–236, DOI: 10.1111/j.1365-2818.1993.tb03346.x (1993).
4. Tomer, R., Khairy, K., Amat, F. & Keller, P. J. Quantitative high-speed imaging of entire developing embryos with simultaneous multiview light-sheet microscopy. *Nat. methods* **9**, 755–63, DOI: 10.1038/nmeth.2062 (2012).
5. Vettenburg, T. *et al.* Light-sheet microscopy using an Airy beam. *Nat. methods* **11**, 541–4, DOI: 10.1038/nmeth.2922 (2014).
6. Huisken, J., Swoger, J., Bene, D., Wittbrodt, J. & Stelzer, E. H. K. Optical Sectioning Deep Inside Live Embryos by Selective Plane Illumination Microscopy. *Science* **305**, 1007–1009, DOI: 10.1126/science.110003 (2004).
7. Corning. Corning® Matrigel® Matrix - Frequently asked questions (2019).
8. Buesa, R. J. Notes and Queries. *Biotech. & Histochem.* **85**, 269–270 (2010).
9. Weiswald, L.-B. *et al.* In situ protein expression in tumour spheres: development of an immunostaining protocol for confocal microscopy. *BMC Cancer* **10**, 106, DOI: 10.1186/1471-2407-10-106 (2010).
10. Jonkman, J., Brown, C. M., Wright, G. D., Anderson, K. I. & North, A. J. Guidance for quantitative confocal microscopy. *Nat. Protoc.* DOI: 10.1038/s41596-020-0307-7 (2020).
11. Melan, M. A. & Sluder, G. Redistribution and differential extraction of soluble proteins in permeabilized cultured cells. Implications for immunofluorescence microscopy. *J. Cell Sci.* **101**, 731 (1992).
12. Smyrek, I. & Stelzer, E. H. K. Quantitative three-dimensional evaluation of immunofluorescence staining for large whole mount spheroids with light sheet microscopy. *Biomed. optics express* **8**, 484–499, DOI: 10.1364/BOE.8.000484 (2017).
