# Supplementary material for: Immunostaining protocol for infiltrating brain cancer spheroids for light-sheet imaging
Source: PLoS One. 2023 Feb 9;18(2):e0281161. doi: 10.1371/journal.pone.0281161 (PMC9910650; doi:10.1371/journal.pone.0281161)
Supplement: Attachment — Submitted filename: Response_PlosONE.pdf [file pone.0281161.s002.pdf]

Dear editor and reviewers,

We are very pleased that the reviewers overall find our protocol to be very useful. However, we greatly appreciate the suggestions and questions from the reviewers, which we used to sharpen our description and improve the reproducibility of our revised protocol.

The manuscript template and the description of the supplementary file has been changed to fit the Plos ONE style. Besides, we have uploaded the requested .io file and included our full ethics statement in the *Methods* section of our manuscript file. Below we address (point-by-point) all questions and comments raised by the reviewers and in the manuscript all changes in the protocol and manuscript are marked with yellow.

Reviewer #1

1. **It would be useful, but may not be strictly necessary to have some quantifiable benchmarks associated with the protocol - what percentage of surface cells were stained using this method, and what percentage of those cells are expected to infiltrate the Matrigel?**

   For the tested cell lines, we found that all (100%) surface cells were stained. However, we recommend using immunolabeling of surface-attached cells or spheroids in liquid media to benchmark. Therefore, we inserted the following paragraph in *Troubleshooting: "Low levels of fluorescence: For diagnostics, immunostain the spheroids while in liquid media following the protocol of section Immunostaining surface-attached cells. The result can be helpful when trying to tune the protocol of section Immunostaining spheroids through hydrogel for proper staining of your tissue sample."*
   We also added the following to the second paragraph of *Troubleshooting*: "*Another parameter to tune in case of low specificity, is the concentration of formaldehyde (fixative). There is a balance between using sufficient amounts of fixative to preserve cellular morphology without reducing the antigenicity [Jonkman et al., Nature Prot. 2020].*
   Also, as antibodies targeting intracellular proteins require permeabilization of the cells' plasma membranes, we used Triton X-100 in a concentration of 0.2% diluted in PBS. However, insufficient permeabilization can hinder antibody penetration, while too high Triton X-100 concentration can lead to the loss or displacement of the target protein [Melan et al., J. Cell Sci. 1992; Smyrek et al., Biomed. Opt. Express 2017]. We therefore suggest varying Triton X-100 concentrations between 0.1-1\% to optimize for your protein of interest."

2. **The speculation that as the cells migrate from the tumor surface, the cells underneath will then repeat the process of vim expression and infiltration is an interesting one, but is this something that the authors have been able to validate in any way? Would this require another round of staining or is this something that could be easily observed in the same**

assay based on the penetration of the vim staining within the spheroid? An answer to this question could be rather impactful in terms of the application of this technique.

This speculation is based on what we learned from label-free invasion assays: Migration seems to start from surface cells or (single cells) that forms long filopodia before migrating [Audoin et al. (2022), Sci Rep 12:14713; Nousi et al. (2021), Biochem Biophys Rep 28:101120] and references in the manuscript. When they detach the cells underneath seem to repeat this. Regarding whether we have been able to validate this, then not yet. We do show in the manuscript that only vim expression is high on the cell surface and zero underneath, in sharp contrast to the nuclear stain (Fig 3C). However, for this to become something more than a qualified guess, more experiments are needed to quantify penetration depth of the antibodies etc.

However, we think that an assay using this protocol has potential to test this hypothesis as i) light-sheet imaging allows deeper imaging compared to other imaging modes, like confocal laser-scanning microscopy and ii) the protocol allows for sequential immunostaining.

3. **The manuscript could use some slight editing for grammar and the protocol has some additional characters (????) that don't influence the method but add some confusion.**

   The question marks were a mistake. This has now been fixed and we also tried to remove grammar mistakes etc.

Reviewer #2

1. **what are those question markers in section "cell culture of primary specimen"**

   A mistake, this is now fixed.

2. **Replace all the "agent" to " reagent"**

   This has now been done.

3. **in the fixation reagent section there is description about tips of how to wash cells. Where in the Procedure section these tips should be applied to? Is in the "Immunostaining spheroids through hydrogel"? However, there are different description in the**

"Immunostaining spheroids through hydrogel" fixation section. Which one the readers should follow? It is really confusing!

As correctly pointed out by the reviewer, this was indeed confusing. We have now removed this information from the description of the fixation and in the *Fixation* subsection of *Immunostaining spheroids through hydrogel,* we inserted another c), such that it now reads: "*Aspirate fixation reagent. NB: As para-formaldehyde liquifies Matrigel, aspiration is difficult to do in one go without harming the sample. Instead add 125 uL washing buffer and aspirate 175 uL (containing diluted paraformaldehyde). Repeat this once again to remove the 100 uL fixation reagent*."

4. **in the Cell Culture section, please specify the number of cells and size of culture dishes. Not just use the description "small culture plate"**

   We have now specified this by rewriting the paragraph: "*4. Resuspend the pellet in 5 ml medium (37 degrees) in a 60 mm cell culture plate to ensure that the cells have time to overcome the stress before being diluted further to ~50% confluency.*

5. **In Gravitation-assisted spheroid formation what is the sentence "The spheroids can be immunofluorescence stained directly using the procedure in section Immunostaining surface-attached cells" meaning?**

   For clarification, this sentence has now been changed to the following paragraph: "*These spheroids can be immunofluorescence stained in two ways. Either when in liquid medium (following the procedure in section Immunostaining surface-attached cells) or after embedment in hydrogel as described in the following*."

6. **In "Imaging spheroids in hydrogel", why the authors put plate on ice to liquefy the hydrogel matrix, isn't matrigel already dissolved in PFA? And can the authors please add a schematic cartoon to show how to set up the imaging tube?**

   We acknowledge that this part was very unclear in the former version of the protocol, to improve we changed the introducing sentence to the following: "*However, this requires that the Matrigel is re-liquefied to allow the displacement of the spheroid to the appropriate imaging sample. For light-sheet imaging we followed these steps:*"

   Furthermore, we inserted the requested cartoon and we decided to add more details to (2): "*This part is tricky, so alternatively spheroids and matrix can be moved from the well to an ice-cold culture dish with a 1 ml pipette (to prevent damaging flow rates), before being aspirated into*

the tube under a microscope, as sketched in Figure 1.. This alternative procedure allows for better localization of the spheroid (or spheroids) in the tube. ”

7. **In "Immunostaining surface-attached cells", why wash the cells with cold PBS, doesn't that detach the cells? And please specify the time of washes.**

   We used cooled PBS as this was recommended by the manufacturer of the antibodies (Novus Biological) and we only now realize that this point is very disputed. In our understanding the arguments are either for minimizing autolysis, for work-flow considerations, or to prevent detachment of cells, as suggested by Reviewer 2. We did not experience any detachment of cells. However, we did not test the difference between adding PBS at room temperature versus cold either.

8. **For primary antibody staining, what time and temperature of incubation the author was using for Vim1? Because in the data part, authors mentioned they have tried 1 hour and 3 hour and no difference was observed. Please make it clear in the protocol and make sure consistent with the data description.**

   This sentence now reads: "*...we tried different reaction times (from 1 hour to 3 hours). We found no obvious differences,...*" As the information in the protocol section *Immunostaining spheroids through hydrogel* - 4 is correct.

9. **For the Data part, in figure 3C, it is hard to understand the way of quantification of the intensity based on the schematic the authors provided.**

   We changed the last part of the caption of Fig 3C to be "*Inset: Sketch of how the intensity distribution over the radial vector is averaged. Intensities are measured for a rotating radial vector in a 4 um slice (10 images) of the spheroid (50 um depth) as detailed in Materials and Methods.*" and for further clarification, we inserted the following sentence in the subsection *Image processing* in *Materials and Methods* (of manuscript): "*This corresponds to averaging over a rotating radial vector, as sketched in the inset of Fig 3*."
